# Oxygen Vacancy Dynamics in Highly Crystalline Zinc Oxide Film Investigated by PIERS Effect

**DOI:** 10.3390/ma14164423

**Published:** 2021-08-07

**Authors:** Grégory Barbillon

**Affiliations:** EPF-Ecole d’Ingénieurs, 92330 Sceaux, France; gregory.barbillon@epf.fr

**Keywords:** PIERS, SERS, zinc oxide, gold, oxygen vacancy

## Abstract

Surface-enhanced Raman spectroscopy (SERS) is commonly employed as an analysis or detection tool of biological and chemical molecules. Recently, an alternative section of the SERS field has appeared, called photo-induced enhanced Raman spectroscopy (PIERS). This PIERS effect is based on the production of the oxygen vacancies (V${}_{0}$) in metal-oxide semiconductor thin-film (or other structures) by irradiation with UV light, thus enabling a Raman signal enhancement of chemical molecules through charge transfer processes between this photo-irradiated semiconductor film (or other structures) and these chemical molecules via metallic nanoparticles deposited on this photo-irradiated substrate. The PIERS technique can enable studying the dynamics of the oxygen vacancies under ambient and operando conditions compared to conventional tools of analysis. In this paper, we present the results obtained on the formation and healing rates of surface oxygen vacancies (V${}_{0}$) in a highly crystalline ZnO film investigated by the PIERS effect, and we compare these results to the literature in order to study the effect of the crystallinity on these formation and healing rates of V${}_{0}$ in a ZnO film.

## 1. Introduction

Surface-enhanced Raman spectroscopy (SERS) is an excellent analytical tool mainly employed in the detection of various chemical or biological molecules [1,2,3,4,5,6,7,8]. In addition, the SERS effect is also very useful for studying the surface catalytic reactions in order to know their mechanism and their intermediate species involved in these reactions [9,10,11,12,13,14,15,16]. In this SERS field, an alternative section has emerged named photo-induced enhanced Raman spectroscopy (PIERS), which gives the possibility of having supplementary chemical enhancement of the Raman signal compared to conventional SERS, by employing a photo-activated semiconductor substrate [17,18,19,20]. This supplementary chemical enhancement is due to the presence of surface oxygen vacancies (V${}_{0}$) created during the pre-irradiation of the semiconductor substrate by UV light [17,18,19,20]. In the literature, it is well-reported that UV light can allow the creation of defects/vacancies in different materials (nanostructured or not), such as metal oxides [17,18,19,20,21,22,23,24], silicon dioxide [25,26], and single-wall carbon nanotubes [27], for instance. In addition, the production of the oxygen vacancies (V${}_{0}$) permits enhancing the Raman signal of chemical molecules through charge transfer processes between the photo-irradiated semiconductor film (or other structures) and these chemical molecules via metallic nanoparticles deposited on this photo-irradiated substrate [17,18,19]. This PIERS effect has already been used for sensitive detection of several molecules such as thiophenol [19,28], explosives [17], pollutants [17] and toxic organics [29]. In addition, this PIERS effect can also be employed for the investigation of the dynamics of these surface oxygen vacancies V${}_{0}$ in metal-oxide semiconductor surfaces. With this technique, excellent information on the dynamics of these oxygen vacancies (V${}_{0}$) can be obtained under ambient and operando conditions compared to more conventional tools of analysis [30,31,32]. Indeed, several works have already investigated the formation of oxygen vacancies by using X-ray photoelectron spectroscopy and electron paramagnetic resonance under ultra-high vacuum conditions and/or at low temperatures [33], and also the migration, healing, and control of the concentration of oxygen vacancies under ambient conditions by employing atomic force microscopy [34], scanning tunneling microscopy [35] and Kelvin probe force microscopy [36]. For all these techniques, specific conditions are necessary, such as ultra-high vacuum, low temperatures, and very flat substrates (e.g., single crystals). Thus, the rates of formation and healing of oxygen vacancies are not immediately observed, especially at ambient conditions. Moreover, to the best of our knowledge, only one study has reported on the dynamics of the oxygen vacancies in metal-oxide semiconductor films investigated by the PIERS technique, where the authors studied thin films of titanium dioxide, tungsten trioxide, and zinc oxide (weakly crystalline for ZnO) [30].

In this paper, we present the results obtained on the formation and healing rates of surface oxygen vacancies (V${}_{0}$) in a highly crystalline ZnO film investigated by the PIERS effect, and we compare these results to the literature in order to study the effect of the crystallinity on the formation and healing rates of V${}_{0}$ for a ZnO film.

## 2. Experimental Details

### 2.1. Fabrications of ZnO Film and Gold Nanoparticles

The zinc oxide ZnO films were grown on c-plane sapphire (c-Al${}_{2}$O${}_{3}$) substrates by pulsed laser deposition from a 99.9995% pure sintered ZnO target using a KrF excimer laser. Then, it was followed by an annealing at 600 ${}^{\circ }$C in O${}_{2}$. For the fabrication of gold nanoparticles, the Turkevich method was used, where 1 mL of trisodium citrate (Na${}_{3}$C${}_{6}$H${}_{5}$O${}_{7}$; concentration of 8.5 × 10${}^{-4}$ M) was added to a boiling aqueous solution of 20 mL of HAuCl${}_{4}$ under conditions of vigorous agitation over 30 min. The average diameter of gold nanoparticles is 30 nm.

### 2.2. Functionalization of Au/ZnO Films with Thiophenol Molecules

The preparation of thiophenol solution was made by dissolution of a thiophenol powder in ethanol at 1 mM concentration. This latter was then diluted with ethanol at 1 μM concentration. Next, the Au/ZnO film was dipped in this solution for 24 h, then dried thoroughly with a nitrogen gun.

### 2.3. Irradiation of Highly Crystalline ZnO Film Decorated with Gold Nanoparticles with UV Light

For the UV-irradiation of the sample, a short-wave UV quartz pencil lamp (Edmund Optics, Lyon, France; 254 nm, 4.89 eV, nominal output of 4.5 mW/cm${}^{2}$) was employed at a distance of 2.4 cm above the sample.

### 2.4. Structural and Raman Characterizations

For the structural characterization of the highly crystalline ZnO film decorated with gold nanoparticles, an X-ray diffraction (XRD) pattern was recorded by using a Siemens D5000 XRD system (Siemens, Erlangen, Germany) in reflection mode with a quartz monochromator (CuK$\alpha_{1}$ = 1.54056 Å). For the Raman measurements, a Labram spectrophotometer from Horiba Scientific (Kyoto, Japan) having a spectral resolution of 1 cm${}^{-1}$ was employed. A laser with a wavelength of 633 nm (power = 1 mW) and an acquisition time of 5 s was employed for these measurements. The laser was focused on the zinc oxide film by employing a microscope objective (×100, N.A. = 0.9). Then, the Raman signal was collected by this same objective in a backscattering configuration. Each time, we recorded fifteen Raman spectra at different positions.

## 3. Results and Discussion

**Figure 1:**
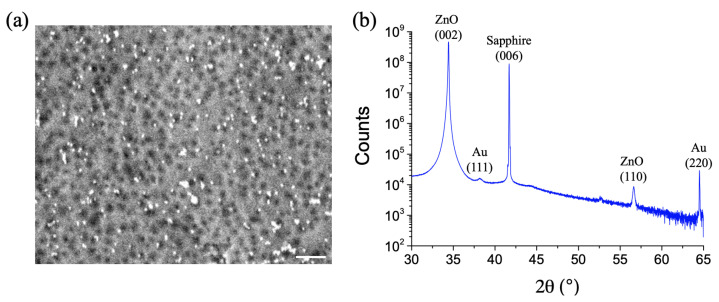
(**a**) SEM image of the highly crystalline ZnO film decorated with gold nanoparticles with a diameter of 30 nm (scale bar = 300 nm). (**b**) X-ray diffraction pattern recorded for the highly crystalline ZnO film with AuNPs on c-Al${}_{2}$0${}_{3}$ substrate (sapphire).

For our study, a highly crystalline ZnO film with a thickness of 200 nm was grown on a sapphire (c-Al${}_{2}$O${}_{3}$) substrate by pulsed laser deposition. Next, gold nanoparticles (AuNPs) with a diameter of 30 nm were drop-casted on the ZnO film, then air-dried (see Figure 1a). From Figure 1b, the X-ray diffraction (XRD) pattern displays a very intense and sharp diffraction peak for the (002) plane and a weak diffraction peak for the (110) plane of ZnO (wurtzite), denoting a high degree of crystallinity. In addition, another intense diffraction peak is displayed corresponding to the (006) plane of the sapphire (c-Al${}_{2}$0${}_{3}$) substrate. Finally, two weak diffraction peaks are also recorded corresponding to the (111) and (220) planes of gold.

**Figure 2:**
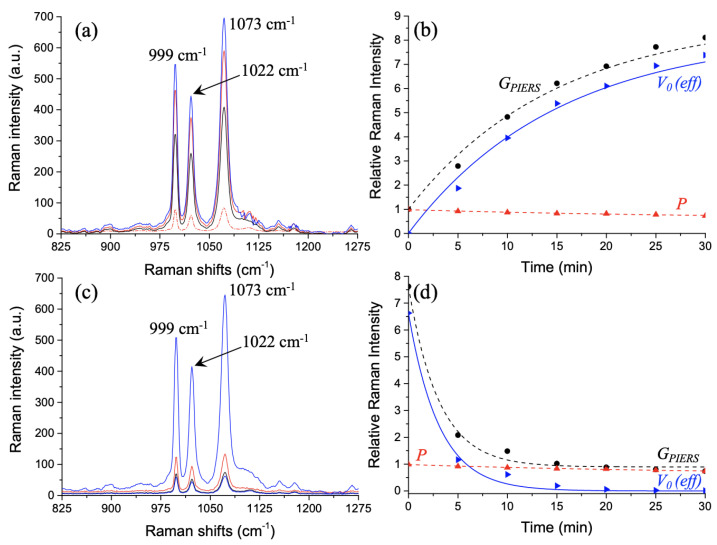
(**a**) Raman spectra for a UV-irradiation time of 0 min (the red dashed line, SERS spectrum serving as a reference), 10 min (in black color, PIERS spectrum), 20 min (in red color, PIERS spectrum) and 30 min (in blue color, PIERS spectrum). (**b**) Variation recorded for the relative Raman intensity of the peak at 1073 cm${}^{-1}$ called $G_{PIERS}$ (experimental points = full black circles; fitting curve (R${}^{2}$ = 0.965) = black dashed curve) under UV irradiation, where $G_{PIERS}$ can be expressed by employing Equation (1), where $V_{0}$(*eff*) corresponds to the effective number of oxygen vacancies (experimental points = blue full triangles; fitting curve (R${}^{2}$ = 0.968) = blue curve), and *P* corresponds to the photobleaching (experimental points = red full triangles; fitting curve (R${}^{2}$ = 0.972) = red dashed curve). (**c**) Raman spectra for a relaxation time of 0 min (in blue color), 10 min (in red color), 20 min (in black color) and 30 min (in dark color) after stopping UV irradiation. (**d**) Variation recorded for the relative Raman intensity of the peak at 1073 cm${}^{-1}$ called $G_{PIERS}$ (with the same correspondence reported in (**b**) for full black circles and black dashed curve (R${}^{2}$ = 0.993)) after UV irradiation, where $G_{PIERS}$ can be expressed by employing Equation (1), where $V_{0}$(*eff*) corresponds to the effective number of oxygen vacancies (with the same correspondence reported in (**b**) for the blue triangles and blue curve (R${}^{2}$ = 0.994)), and *P* corresponds to the photobleaching (with the same correspondence reported in (**b**) for the red triangles and red dashed curve (R${}^{2}$ = 0.972)).

For our investigation, the gold nanoparticles were functionalized with thiophenol molecules following the protocol described in Section 2.2. Next, we recorded a SERS spectrum used as a reference, where three Raman peaks are displayed corresponding to characteristic peaks of thiophenol molecules at 999, 1022, and 1073 cm${}^{-1}$ [37,38] (see Figure 2a: the red dashed line). Respectively, these peaks are attributed to C–H out-of-plane bending and ring out-of-plane deformation (called: γ(CH) and r–o–d), then to the ring in-plane deformation and C–C symmetric stretching (called: r–i–d and ν(CC)), and finally to the C–C symmetric stretching and C–S stretching (called: ν(CC) and ν(CS)). Throughout our study, we used the Raman peak at 1073 cm${}^{-1}$ for determining the PIERS gain.

Afterward, the AuNP/ZnO film was irradiated in 5 min steps with the UV light, and Raman spectra were recorded with the UV lamp off. This process was renewed for each step of 5 min up to 30 min. For all of the Raman characterization, the sample position was fixed, and the UV light was switched off. For the process of UV-irradiation, the laser source used for Raman characterization was blocked by using a shutter. From Figure 2a, we observed an increase in Raman band intensities beyond the SERS intensities during and just after the UV irradiation. We have assigned the enhancement of the relative Raman intensity (ratio of the PIERS intensity on SERS intensity) to the formation of oxygen vacancies in the PIERS effect [17,19]. This enhancement in the relative Raman intensity called $G_{PIERS}$ (defined as the ratio $I_{PIERS}/I_{SERS}$) can be expressed as follows (Equation (1)) [30]:

(1) $$\begin{array}{c} G_{PIERS}=V_{0}\left(\mathrm{eff}\right)+P \end{array}$$

where $V_{0}$(*eff*) represents the effective number of oxygen vacancies resulting from the difference between the formed and healed numbers of oxygen vacancies. *P* represents the photobleaching of thiophenol molecules under the Raman laser illumination on non-irradiated samples. Each of the three variables of Equation (1) is related to the variations in Raman band intensity, which are dependent on time. Figure 2b displays three variables described above during the UV irradiation. *P* has been evaluated by measuring the SERS intensity for different exposure durations to the Raman excitation laser and by dividing it by the SERS intensity obtained at the initial time (t = 0 min) (see the red dashed curve in Figure 2b). $V_{0}$(*eff*) has been deduced by subtracting *P* from $G_{PIERS}$ (see the blue curve in Figure 2b).

**Figure 3:**
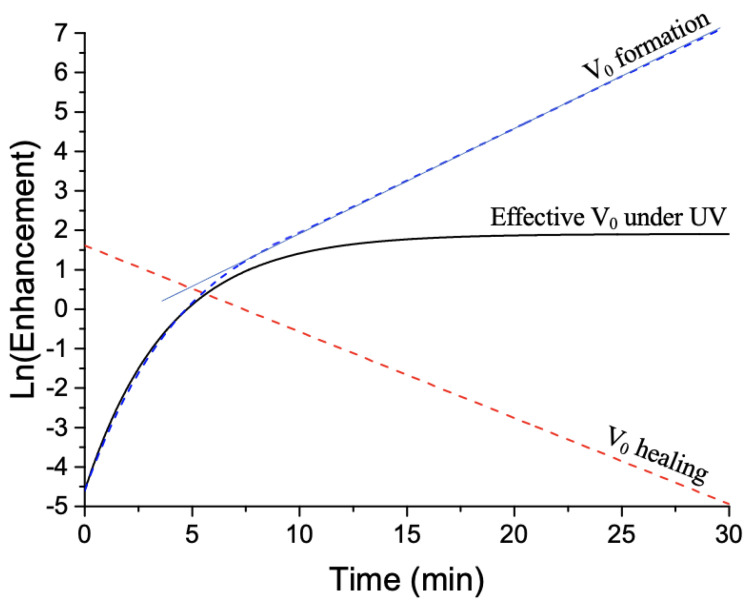
Logarithm of enhancement ($G_{PIERS}$−*P*) owing to oxygen vacancies as a function of time. The red dashed line is obtained from the blue curve $V_{0}$(*eff*) of Figure 2d, corresponding only to the $V_{0}$ healing and calculated by Equation (1). The black curve is obtained from the blue curve $V_{0}$(*eff*) of Figure 2b, corresponding to the effective number of oxygen vacancies resulting from the difference between the formed and healed numbers of $V_{0}$. The blue dashed curve is obtained through the deconvolution of curves of effective $V_{0}$ (under UV) and $V_{0}$ healing by using Equation (2). The values of each rate (formation and healing) correspond to the slope of the linear region of corresponding curves reported in Table 1.

For the PIERS decay (relaxation mechanism) after UV irradiation of 90 min, we recorded a Raman spectrum in 5 min steps upon exposure to air, and we observed a decreasing of Raman intensity down to a value close to that recorded for the SERS spectrum serving as a reference (see Figure 2c). Thus, the decay in the relative Raman intensity (see Figure 2d) is assigned to the healing of the oxygen vacancies in the PIERS effect [17,19]. As previously, $V_{0}$(*eff*) has been deduced by subtracting *P* from $G_{PIERS}$ (see the blue curve in Figure 2d), where *P* has already been determined previously and displayed in Figure 2d (see the red dashed curve). In addition, we have fitted the experimental results of photobleaching *P* with a simple exponential decay (form: $Ae^{-Bt}+C$ where *A*, *B*, and *C* are fit parameters where B corresponds to a rate related to the decay time constant), which is well-known for describing the rate of molecule photobleaching under the excitation laser during the Raman characterization [30,39]. The experimental results of $V_{0}$(*eff*) have been fitted with the same form of the exponential function described previously (form: $Ae^{-Bt}+C$ where *B* corresponds to the rate of each process) and corresponding to the integrated rate law of first-order reactions for the experience after UV irradiation (see Figure 2d) and a pseudo-first-order rate equation for the experience under UV irradiation (see Figure 2b). In order to determine the formation and healing rates of oxygen vacancies, we have plotted the logarithm of enhancement ($G_{PIERS}$−*P*) as a function of time (see Figure 3). In Figure 3, the black curve is obtained from the blue curve $V_{0}$(*eff*) of Figure 2b, corresponding to the effective number of oxygen vacancies resulting from the difference between the formed and healed numbers of $V_{0}$. Moreover, the rate of the effective number of oxygen vacancies can be expressed as follows (Equation (2)) [30]:

(2) $$\begin{array}{c} \frac{dV_{0}\left(\mathrm{eff}\right)}{dt}=\frac{dV_{0form}}{dt}+\frac{dV_{0heal}}{dt} \end{array}$$

where $dV_{0form}/dt$ and $dV_{0heal}/dt$ correspond to the formation and healing rates of oxygen vacancies, respectively. Next, the red dashed line is obtained from the blue curve $V_{0}$(*eff*) of Figure 2d, corresponding only to the $V_{0}$ healing and calculated by Equation (1). In this case, $dV_{0}\left(\mathrm{eff}\right)/dt$ = $dV_{0heal}/dt$ because no additional UV irradiation is realized, inducing no additional $V_{0}$ vacancy ($dV_{0form}/dt$ = 0). Thus, $V_{0heal}$ = $G_{PIERS}$−*P*, and the rate of $V_{0}$ healing is determined by measuring the slope of the red dashed line and reported in Table 1.

Finally, the blue dashed curve is obtained through the deconvolution of curves of effective $V_{0}$ (under UV) and $V_{0}$ healing by using Equation (2). The rate of $V_{0}$ formation is determined by measuring the slope of the linear region of the blue dashed curve and reported in Table 1.

By comparing our results to the literature (see Table 1), we observed that the rates of formation and healing for oxygen vacancies $V_{0}$ were higher for a highly crystalline ZnO film than for a weakly crystalline ZnO film. We assumed that was due to the porous structure of our ZnO film compared to the smooth structure of the ZnO film observed in Reference [30], which is mentioned for reducing the vacancy formation.

## 4. Conclusions

In this paper, we determined the formation and healing rates of surface oxygen vacancies (V${}_{0}$) in a highly crystalline ZnO film with the PIERS technique, and we observed an effect of the crystallinity in a ZnO film on these rates by comparing them with the literature. We found that these rates were higher for a highly crystalline ZnO film than for a weakly crystalline ZnO film due to the porous structure of the highly crystalline ZnO film. In addition, thanks to the PIERS technique, probing surface oxygen vacancies (V${}_{0}$) is possible in real-time conditions compared to other existing techniques. Another advantage of using this PIERS technique for studying oxygen vacancies is its capability of measurements in operando conditions. Finally, the PIERS technique can allow probing macroscopic zones in a short time (a couple of seconds or minutes) compared to scanning probe techniques, where only a nanoscale zone can be probed under high vacuum conditions, whose measurement can take several hours. Thus, the PIERS technique can permit the understanding of vacancy states in catalytic materials and other metal oxides in a non-destructive way. 

## Figures and Tables

**Table 1:** $V_{0}$ formation and healing rates calculated from changes of the Raman band enhancement ($G_{PIERS}$−*P*) over time for two zinc oxide films with a different crystallinity (weakly crystalline film = all the XRD peaks are weakly intense (see reference [30]); highly crystalline film = some XRD peaks are very intense (see Figure 1b)).

Sample	Weakly Crystalline ZnO Film	Highly Crystalline ZnO Film
$V_{0}$ formation rate	0.254 ± 0.108 min${}^{-1}$	0.272 ± 0.008 min${}^{-1}$
$V_{0}$ healing rate	0.196 ± 0.021 min${}^{-1}$	0.206 ± 0.009 min${}^{-1}$
References	[30]	This work

## Data Availability

Data is contained within the article.
